# Antitumor Activity of Lenvatinib (E7080): An Angiogenesis Inhibitor That Targets Multiple Receptor Tyrosine Kinases in Preclinical Human Thyroid Cancer Models

**DOI:** 10.1155/2014/638747

**Published:** 2014-09-10

**Authors:** Osamu Tohyama, Junji Matsui, Kotaro Kodama, Naoko Hata-Sugi, Takayuki Kimura, Kiyoshi Okamoto, Yukinori Minoshima, Masao Iwata, Yasuhiro Funahashi

**Affiliations:** ^1^Biomarkers and Personalized Medicine Core Function Unit, Eisai Co., Ltd, Tsukuba, Ibaraki 300-2635, Japan; ^2^Discovery Biology, Oncology Product Creation Unit, Eisai Co., Ltd, Tsukuba, Ibaraki 300-2635, Japan; ^3^Biomarkers and Personalized Medicine Core Function Unit, Eisai Inc., 4 Corporate Drive, Andover, MA 01810, USA

## Abstract

Inhibition of tumor angiogenesis by blockading the vascular endothelial growth factor (VEGF) signaling pathway is a promising therapeutic strategy for thyroid cancer. Lenvatinib mesilate (lenvatinib) is a potent inhibitor of VEGF receptors (VEGFR1–3) and other prooncogenic and prooncogenic receptor tyrosine kinases, including fibroblast growth factor receptors (FGFR1–4), platelet derived growth factor receptor *α* (PDGFR*α*), KIT, and RET. We examined the antitumor activity of lenvatinib against human thyroid cancer xenograft models in nude mice. Orally administered lenvatinib showed significant antitumor activity in 5 differentiated thyroid cancer (DTC), 5 anaplastic thyroid cancer (ATC), and 1 medullary thyroid cancer (MTC) xenograft models. Lenvatinib also showed antiangiogenesis activity against 5 DTC and 5 ATC xenografts, while lenvatinib showed in vitro antiproliferative activity against only 2 of 11 thyroid cancer cell lines: that is, RO82-W-1 and TT cells. Western blot analysis showed that cultured RO82-W-1 cells overexpressed FGFR1 and that lenvatinib inhibited the phosphorylation of FGFR1 and its downstream effector FRS2. Lenvatinib also inhibited the phosphorylation of RET with the activated mutation C634W in TT cells. These data demonstrate that lenvatinib provides antitumor activity mainly via angiogenesis inhibition but also inhibits FGFR and RET signaling pathway in preclinical human thyroid cancer models.

## 1. Introduction

Thyroid cancer is a common malignant endocrine tumor, the incidence of which has recently been increasing. More than 90% of thyroid cancers are the follicular or papillary types known as differentiated thyroid cancer (DTC) [1], which comprises 1% of all cancers globally. Ninety percent of patients with DTC survive at least 10 years [2]; however, DTC patients with radioactive iodine- (RAI-) refractory disease have a median survival of 2.5 to 3.5 years from detection of distant metastases [3, 4]. The cytotoxic agents used to treat RAI refractory disease have been universally classified as having only marginal efficacy and substantial toxicity [5, 6]. Approximately 10% of all thyroid cancers are medullary thyroid cancer (MTC) [7], which is a distinct C-cell tumor of the thyroid. MTC occurs in both sporadic (75% of patients) and hereditary settings, and the latter includes three distinct inherited cancer syndromes: familial MTC, multiple endocrine neoplasia type 2A (MEN2A), and MEN2B [8]. MTC is associated with a favorable prognosis (i.e., 10-year survival rate, ~70%) in the case that the disease is treated at an early stage, but the prognosis is poor (i.e., 10-year survival rate, <50%) in patients with distant metastatic disease [9]. Anaplastic thyroid cancer (ATC) remains one of the most deadly human diseases but accounts for fewer than 2% of all thyroid cancers [10, 11]. Average survival after diagnosis is only 4 to 6 months [10, 12], and the tumor is usually well advanced by the time of diagnosis. ATC tumors are generally resistant to chemotherapy, and currently there is no effective therapeutic regimen for the treatment of ATC.

Various genetic alterations participate in the tumorigenesis of thyroid cancer [13]. BRAF V600E is one of the most frequent mutations, occurring in approximately 45% of papillary thyroid cancers (PTCs) [14] and 26% of ATCs [14, 15]. Other genetic alterations in PTC are point mutations in RAS [13, 16] and rearrangement of RET [17]. In follicular thyroid carcinoma (FTC), mutations in RAS [18, 19] and rearrangement of the PPAR*γ* and PAX8 genes [18] are also common. Germline mutations in the RET proto-oncogene cause hereditary MTC [20–22], and approximately 50% of patients with sporadic MTC have somatic RET mutations [23, 24]. Accordingly, new molecularly targeted therapies are needed for the treatment of thyroid cancer.

Tumor angiogenesis—the formation of blood vessels within tumors—plays a key role in cancer cell survival, local tumor growth, and the development of distant metastases [25–28]. Numerous angiogenic factors have been identified, including VEGF, basic fibroblast growth factor (bFGF), hepatocyte growth factor, interleukin-8, and PDGF. VEGF is a crucial regulator of both physiologic and pathologic angiogenesis via its binding to the cognate receptor VEGFR2. Since increased VEGF expression is significantly associated with advanced-stage thyroid cancer [29], the use of inhibitors against VEGFR2 signaling pathway may represent a viable approach to controlling malignant thyroid cancer [30]. Inhibitors of the VEGFR2 signaling pathway show antitumor activity against various types of tumors, and consequently many of them are now in clinical use [31]. In phase 2 clinical trials of thyroid cancers motesanib, axitinib, and pazopanib, which are multiple receptor-tyrosine kinase inhibitors, have shown promising antitumor activity [32]. The US FDA recently approved sorafenib for the treatment of patients with locally recurrent or metastatic, progressive DTC that is refractory to RAI treatment [33]. Vandetanib [34] and cabozantinib have been approved for use in the treatment of advanced or metastatic MTC. Therefore, molecularly targeted agents for the VEGFR2 signaling pathway are expected as new thyroid cancer therapy. Beside VEGFR2 signaling pathway, other receptor tyrosine kinases (RTKs) have significant roles in thyroid cancer. Overexpression of fibroblast growth factor receptor [35] and mutations of RET [23, 24] are reported to participate in the development and aggressive phenotypes of thyroid cancers. Given that the most of VEGFR2 inhibitors which target multiple receptors, inhibition of those RTKs could improve thyroid cancer therapy in addition to targeting the VEGFR2 signaling pathway.

Lenvatinib mesilate (lenvatinib) inhibits the multiple RTKs that target VEGF receptors (VEGFR1–3), FGF receptors (FGFR1–4), PDGF receptor *α* (PDGFR*α*), KIT, and RET [36]. Besides its antiangiogenesis activity based on VEGFR2 inhibition [36], lenvatinib has also shown antitumor activity based on inhibition of RET phosphorylation in PTC carrying RET/PCT fusion genes [37]. Lenvatinib has shown antitumor activity against multiple tumor types, such as DTC, MTC, melanoma, endometrial and hepatocellular [38, 39]. The purpose of this study was to assess the antitumor activity of lenvatinib and explore its mode of action in preclinical human thyroid cancer models, using DTC, MTC, and ATC cell lines. Here, we describe how lenvatinib inhibited in vivo tumor growth and tumor-induced angiogenesis in various human thyroid cancer xenograft models. We also show that lenvatinib directly inhibits the in vitro proliferation of thyroid cancer cell lines carrying mutations that activate RET or in which FGFR1 is overexpressed.

## 2. Materials and Methods

### 2.1. Compounds

Lenvatinib mesilate (lenvatinib), PD166866, PD173074, imatinib, and Ki6783 were synthesized at Eisai Co., Ltd. (Ibaraki, Japan). Sorafenib was purchased from Bayer (Tokyo, Japan).

### 2.2. Cell Free Kinase Inhibition Assay

The kinase inhibitory activities of lenvatinib and sorafenib against 66 purified recombinant protein kinases (including tyrosine kinases and serine threonine kinases) were examined by using an ELISA and an Off-Chip Mobility Shift Assay (MSA) from Carna Biosciences, Inc. (Kobe, Japan). Briefly, each test compound was mixed with enzyme, substrate, ATP, and Mg under appropriate buffer conditions for the ELISA or MSA. The readout value of the reaction control (complete reaction mixture) was set as 0% inhibition, and the readout value of the background (Enzyme (−)) was set as 100% inhibition; the percent inhibition of each test solution was then calculated. IC_50_ values (the half maximal inhibitory concentration) were calculated from concentrations versus % inhibition curves.

### 2.3. Cells

The human DTC cell lines K1, FTC-133, FTC-236, FTC-238, and RO82-W-1 and the human thyroid follicular epithelial cell line Nthy-ori 3-1 were obtained from DS Pharma Biomedical Co., Ltd. (Osaka, Japan). The human MTC cell line TT was obtained from the American Type Culture Collection (Manassas, VA). The human ATC cell lines 8305C, 8505C, HTC/C3, KHM-5M, and TCO-1 were obtained from the Japanese Collection of Research Bioresources Cell Bank (Osaka, Japan). K1 and RO82-W-1 cells were cultured in a mixture of Dulbecco's Modified Eagle Medium (DMEM), Ham's F12 medium, and MCDB 105 medium (2 : 1 : 1, v/v/v) with 10% FBS; FTC-133, FTC-236, and FTC-238 cells were cultured in a mixture of DMEM and Ham's F12 medium (1 : 1, v/v) with 10% FBS, Nthy-ori 3-1 cells were cultured in RPMI-1640 medium with 10% FBS, TT cells were cultured in RPMI-1640 medium with 15% FBS, TCO-1 cells were cultured in DMEM with 10% FCS, and 8305C and 8505C cells were cultured in Eagle's minimal essential medium (EMEM) supplemented with 10% FBS. KHM-5M cells were cultured in RPMI-1640 medium with 15% FBS. HTC/C3 cells were cultured in DMEM supplemented with 4,500 mg/L glucose and 10% FBS. All cells were grown at 5% CO_2_ and 37°C. DMEM, EMEM, Ham's F12 medium, and RPMI-1640 medium were purchased from Wako (Osaka, Japan). MCDB 105 medium was purchased from Sigma-Aldrich (St. Louis, MO).

### 2.4. Animals

Nude mice (CAnN.Cg-Foxn1nu/CrlCrlj, female, 5-6 weeks old) were obtained from Charles River Laboratories Japan (Kanagawa, Japan). Mice were maintained under specific pathogen-free conditions and housed in barrier facilities on a 12 h light/dark cycle, with food and water ad libitum. All procedures using laboratory animals were done in accordance with all applicable institutional and government regulatory guidelines and policies and were performed in an animal facility accredited by the Center for Accreditation of Laboratory Animal Care of the Japan Health Sciences Foundation.

### 2.5. Human Thyroid Cancer Xenograft Models

Tumor cells were cultured in appropriate culture mediums. Cells were harvested with 0.05% or 0.25% (w/v) trypsin/EDTA and suspended with 50% (v/v) BD Matrigel (BD Biosciences, San Jose, CA) in the mixture of culture mediums at a density of 5–10 × 10^7^ cells/mL. Then, 0.1-0.2 mL of the cell suspension was inoculated subcutaneously into the right flank region of each mouse. When the tumor volume reached between 100 and 300 mm^3^, mice were selected based on their tumor volumes, tumor shapes, physical condition, and body weights to be randomly split into each treatment group: vehicle, lenvatinib, PD173074, or sorafenib (*n* = 5 per group). Lenvatinib, PD173074, and sorafenib were dissolved in sterile distilled water, sterile distilled water containing equimolar hydrochloric acid, and distilled water containing 12.5% (v/v) ethanol and 12.5% (v/v) Cremophor EL, respectively, and administered orally once daily. The tumor size was measured in two dimensions by using a caliper, and the volume was calculated by using the formula: tumor volume (mm^3^) = 1/2 length (mm) × [width (mm)]^2^. The change in tumor volume in the treated group relative to that in the control group was calculated according to the following formula: Δ*T*/*C* = (Δ*T*/Δ*C*) × 100%, where Δ*T* and Δ*C* are the change in tumor volume (i.e., growth) for the treated and vehicle control group, respectively. The percentage of tumor growth inhibition (%TGI) was calculated from the formula: [(1 − Δ*T*/Δ*C*) × 100].

### 2.6. Quantitative RT-PCR

Cells (5 × 10^6^) were seeded and cultured in 6-well culture plates. After overnight culture, total RNA was isolated from the cultured cells by using an RNeasy Mini Kit (Qiagen, Hilden, Germany) according to the manufacturer's protocol. Reverse transcription was carried out with purified RNA by using a High Capacity cDNA Reverse Transcription Kit (Life Technologies, Carlsbad, CA). Synthesized cDNA was used as a template for quantitative polymerase chain reaction (PCR) assays using TaqMan Universal PCR Master Mix (Life Technologies), AmpErase UNG (Life Technologies), and TaqMan probes [*FGFR1* (Hs 00241111),* FGFR2* (Hs 01552926),* FGFR3* (Hs 00179829),* FGFR4* (Hs 00242558),* VEGFR1* (Hs 01904119),* VEGFR2* (Hs 00176676),* VEGFR3* (Hs 01047687),* KIT* (Hs 00174029),* EGFR* (Hs 00193306),* PDGFRA* (Hs 00183486),* PDGFRB* (Hs 00182163),* MET* (Hs 01565580),* RET* (Hs 01120032),* 18S rRNA* (Hs 99999901)] (Life Technologies, Carlsbad, CA) in an ABI 7900 PCR system (Life Technologies). A standard curve was used to determine PCR efficiency. Cycle threshold (Ct) values were determined by using SDS software (Life Technologies). Relative gene expression was normalized to a housekeeping gene (18S rRNA).

### 2.7. Plasmid Construction

The human full-length KIF5B-RET gene [37] was chemically synthesized by GenScript Corp. (Piscataway, NJ) and then amplified by polymerase chain reaction (PCR) using a primer set containing attB recombination sequences. ENTRY vectors for the Gateway cloning system (Life Technologies) were generated via the BP Clonase reaction using the PCR products and the plasmid pDONR221. The expression vector pCLxIP KIF5B-RET was generated via the LR Clonase reaction between each ENTRY vector and the destination vector pCLxIP-DEST [37]. Expression vectors for KIF5B-RET M918T were generated by introducing a point mutation into the pCLxIP KIF5B-RET expression vector.

### 2.8. Western Blot Analysis

Cells (1 × 10^5^ to 3 × 10^6^) were seeded and cultured to subconfluency in 6-well, 100mm, or 150mm cell culture plates in appropriate culture mediums overnight. RO82-W-1 cells were lysed in RIPA buffer (50 mM HEPES [pH 7.4], 150 mM NaCl, 1.5 mM MgCl_2_, 10% [v/v] glycerol, 1% [v/v] Triton X-100, EDTA-free Protease Inhibitor Cocktail (Roche, Mannheim, Germany), Phosphatase Inhibitor Cocktail 2 (Sigma-Aldrich), and Phosphatase Inhibitor Cocktail 3 (Sigma-Aldrich)). TT cells were lysed in lysis buffer (50 mM HEPES [pH 7.4], 150 mM NaCl, 1 mM MgCl_2_, 10% [v/v] glycerol, 1% [v/v] Triton X-100, 1 mM EDTA [pH 8.0], 100 mM NaF, 1 mM phenylmethylsulfonyl fluoride, 1 mM sodium orthovanadate, 10 *μ*g/mL aprotinin, 50 *μ*g/mL leupeptin, and 1 *μ*g/mL pepstatin A). RO82-W-1 cells were starved overnight in culture medium containing 0.5% (w/v) bovine serum albumin (BSA), treated with PD173074, lenvatinib, or sorafenib for 1 h at the indicated concentrations and then stimulated for 10 min with bFGF (20 ng/mL; R&D Systems, Minneapolis, MN) and heparin (Sigma-Aldrich) before being lysed. Cultured TT cells were treated with lenvatinib for 1 h at the indicated concentrations before being lysed. Nthy-ori 3-1 cells were transfected with either KIF5B-RET- or KIF5B-RET M918T-expressing plasmids by using X-tremeGENE9 (Roche Diagnostics K. K., Tokyo, Japan). The next day, the transfected cells were treated with lenvatinib for 1 h before being lysed. Lysates from RO82-W-1 cells were immunoprecipitated with an anti-FGFR1 monoclonal antibody (WH0002260M3[5E9], Sigma-Aldrich). Nude mice bearing RO82-W-1 or TT xenografts were treated once orally with either vehicle or lenvatinib at 3, 10, 30, and 100 mg/kg. Nude mice bearing RO82-W-1 were also treated with sorafenib at 100 mg/kg. Tumors were collected 2 h after administration for RO82-W-1 xenografts and 2, 8, 12, and 24 h after administration for TT xenografts and were then lysed with RIPA buffer or lysis buffer, respectively. Lysed RO82-W-1 xenografts treated with lenvatinib at 30 and 100 mg/kg and sorafenib were immunoprecipitated with an anti-FRS2-*α* polyclonal antibody (FRS2, AF4069; R&D Systems). Immunoprecipitated samples and lysed samples (20–30 *μ*g of protein in 10 *μ*L) were electrophoresed in 5%–20% or 4%–20% (for RET detection) polyacrylamide gels. Separated proteins were transferred onto PVDF membranes (Millipore, Bedford, MA) or Hybond-P (GE Healthcare Life Sciences, Uppsala, Sweden) and the membranes were incubated with the following primary antibodies: FGFR1 (ab76464; abcam, Cambridge, MA), FGFR1 (#3472; Cell Signaling, Beverly, MA) for immunoprecipitated samples, FGFR2 (MAB6842; R&D systems), FGFR3 (#4574; Cell Signaling), FGFR4 (SC-136988; Santa Cruz, Dallas, TX), phospho-FGFR1 (SC-30262R; Santa Cruz), FRS2-*α* (FRS2, SC-8318; Santa Cruz) for cell lysates, FRS2-*α* (FRS2, AF4069; R&D Systems) for tumor tissue lysates, phospho-FRS2-*α* [Thr196] (phospho-FRS2, #3864; Cell Signaling), RET (sc-1290; Sigma-Aldrich), phospho-RET (#3221, Cell Signaling), MEK1/2 (#9122, Cell Signaling), phospho-MEK [Ser217/221] (#9121, Cell Signaling), ERK1/2 (#9102, Cell Signaling), and phospho-ERK1/2 (#9101, Cell Signaling). Blots were detected with an ECL Prime Western Blotting Detection System (GE Healthcare Life Sciences) or with a SuperSignal Enhanced Chemiluminescence Kit (Pierce, Rockford, IL). Immunoreactive bands were visualized by using an LAS-4000 luminescent image analyzer (Fuji Film, Tokyo, Japan), Image Master (GE Healthcare Life Sciences), or Chemi Doc XRS (BioRad, Hercules, CA).

### 2.9. Antiproliferation Assay

Cells (1,000–3,000/well) were seeded and cultured in 96-well culture plates. After overnight culture, the cells were incubated with various concentrations of compounds for 3 days (10 days for TT cells). Cell numbers were determined by using WST-8 (Dojindo, Kumamoto, Japan); 10 *μ*L of WST-8 was added to each well, and absorbance was measured at a wavelength of 450 nm and compared with a reference measurement at 660 or 665 nm by using an MTP-500 microplate reader (Corona Electric, Ibaraki, Japan) or an EnVision Multilabel Reader (Perkin Elmer, Turku, Finland). IC_50_ were graphically obtained from the dose-response curves.

### 2.10. Immunohistochemical Analysis

Tumor tissues were resected from nude mice, embedded in Optimal Cutting Temperature (OCT) compound (Sakura Finetek Japan, Tokyo, Japan), frozen in dry ice-acetone, and then sectioned (8 *μ*m), or they were embedded in paraffin, fixed overnight in immunohistochemical (IHC) Zinc Fixative (BD Biosciences), and then sectioned (6 *μ*m). Tumor microvessels were stained by the indirect immunoperoxidase method with a rat anti-mouse CD31 monoclonal antibody (clone MEC13.3, BD Biosciences) and then visualized by using Vectastain ABC (Dako, Tokyo, Japan). Pericyte was stained by the indirect alkaline phosphatase method with an anti-*α* smooth muscle actin (SMA) antibody conjugated alkaline phosphatase (A 5691, Sigma-Aldrich) and visualized by using Simple Stain AP(R) (Nichirei Biosciences Inc, Tokyo, Japan). Adjacent sections were stained with Hematoxylin-Eosin. Microvessel density (MVD) was determined as described previously [40]. Each section was scanned by using a microscope (VANOX AHBS3, Olympus, Tokyo, Japan) at low magnification to identify 5 areas with the highest densities of CD31-stained microvessels. The microvessels in the selected areas were then counted at 400x magnification. MVD was recorded as the number of microvessels per mm^2^. In the DTC xenografts, the percentage of pericyte-covered microvessels was analyzed. Pericyte-covered vessels were identified by examining the colocalization of CD31- (endothelial cells-) positive vessels and *α*SMA- (pericytes-) positive cells. The number of pericyte-covered vessels was counted in the selected area and then compared with the total number of CD31-stained microvessels in the same area. The percentage of pericyte-covered microvessels was calculated as the number of pericyte-covered microvessels/total number of microvessels × 100 in the selected area. The MVD in the ATC xenograft models was determined by using the digital pathology system ScanScope XT (Aperio, Vista, CA). Briefly, CD31-stained whole tissue slices were scanned on the ScanScope system (Aperio) to generate high-resolution digital slides. By using a digital slide viewer, Aperio ImageScope (v10.0.36.1805, Aperio), 5 regions of interest (ROIs: each 500 *μ*m square) were manually selected as areas with the highest densities of CD31-stained microvessels in each tumor slice. The number of microvessels in each ROI was measured by using Microvessel Analysis Algorithm v1.0 (Aperio), which automatically detects and quantifies microvessels on slides stained with endothelial markers. MVD was expressed as the number of microvessels per mm^2^. The mean MVD of 5 ROIs was defined as the MVD of the tumor. The percentage of microvessel inhibition (%MVI) was calculated from the microvessel density (MVD) according to the following formula: [(1 − MVD of tumor in each compound-treated animal/Mean MVD of tumors in the vehicle control-treated group) × 100].

### 2.11. Gene Mutation Analysis

Cells (2 × 10^6^) were seeded and cultured in 6-well culture plates. After overnight culture, genomic DNA was isolated from the cultured cells (K1, RO82-W-1, FTC-133, FTC-236, and FTC-238) by using the DNeasy Blood & Tissue kit (Qiagen). Mutation analysis for 443 mutations among 32 genes [*ABL1*,* AKT1*,* AKT2*,* APC*,* BRAF*,* CDK4*,* CDKN2A*,* CSF1R*,* CTNNB1*,* EGFR*,* FGFR1*,* FGFR3*,* FLT3*,* HRAS*,* JAK2*,* JAK3*,* KIT*,* KRAS*,* MET*,* MLH1*,* NRAS*,* P53*,* PDGFRA*,* PIK3CA*,* PTEN*,* RB1*,* RET*,* SRC*,* STK11*,* VH1*] was performed by using the MassARRAY System (Sequenom, San Diego, CA) with OncoCarta Panel versions 1.0 and 3.0.

### 2.12. Statistical Analysis

All statistical analyses were performed by using GraphPad Prism 6.0 Software (GraphPad Software, Inc., La Jolla, CA). The significance of the differences between the vehicle and treated groups in terms of both antitumor and antiangiogenesis activity in the human tumor xenograft models was determined by using Dunnett's multiple comparison test. Results were considered significant at *P* < 0.05. To analyze the relationship between antitumor activity (%TGI) and antivascular activity (%MVI), average %TGI and %MVI values were plotted on *X*- and *Y*-axes, respectively. Analysis of covariance (ANCOVA) for %TGI was examined by using %MVI as the covariate.

## 3. Results

### 3.1. Antitumor Activity of Lenvatinib in Human Thyroid Cancer Xenograft Models in Nude Mice

**Figure 1:**
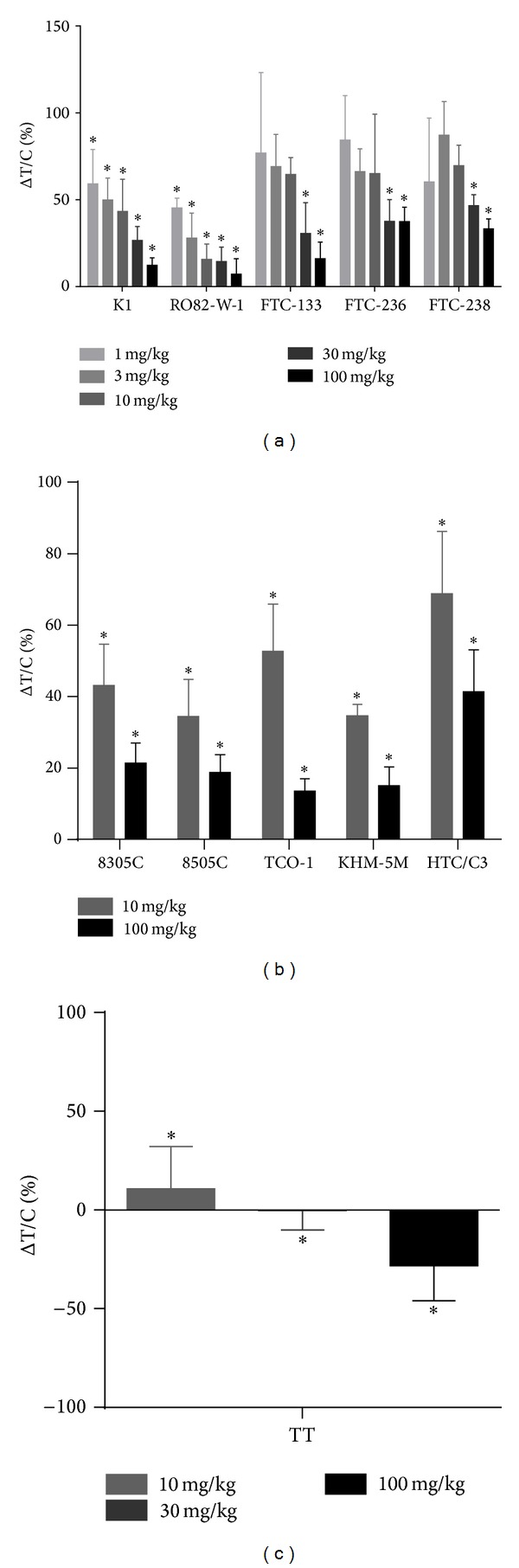
Antitumor activity of lenvatinib in human thyroid cancer xenograft models in nude mice. Nude mice bearing tumor xenografts were treated orally once daily with either vehicle or lenvatinib at the indicated doses when tumor volumes reached between 100 and 300 mm^3^ (day 1). Each group consisted of 5 mice. The change in tumor volume in the treated group relative to that in the control group Δ*T*/*C* (%) was calculated as (Δ*T*/Δ*C*) × 100%, where Δ*T* and Δ*C* are the change in tumor volume for the treated and vehicle control group, respectively. (a) DTC xenograft models. Δ*T*/*C* on day 15. (b) ATC xenograft models. Δ*T*/*C* on day 15. (c) TT xenograft model. Δ*T*/*C* on day 29. Data are shown as means ± SD. *P < 0.05 compared with vehicle-treated mice.

We examined the antitumor activity of lenvatinib in 11 human thyroid cancer xenograft models with 3 types of histology, such as DTC, MTC, and ATC. Five DTC cell lines [1 papillary thyroid cancer line (K1) and 4 follicular thyroid cancer cell lines (RO82-W-1, FTC-133, FTC-236, and FTC-238)], 1 MTC cell line (TT), and 5 ATC cell lines (8305C, 8505C, TCO-1, KHM-5M, and HTC/C3) were examined. Tumor cells were subcutaneously inoculated into the hind flank region of nude mice. Tumors were allowed to grow to sizes of between 100 and 300 mm^3^ before initiation of daily oral treatment with lenvatinib for 14 days (TT cells were treated for 29 days). During treatment with lenvatinib, no macroscopic changes or loss of body weight were observed (data not shown). Lenvatinib showed significant antitumor activity at 30 and 100 mg/kg in all 5 DTC xenograft models and at lower doses (1, 3, and 10 mg/kg) in the K1 and RO82-W-1 xenograft models (Figure 1(a), Supplementary Figure S1(a) in the Supplementary Material available online at http://dx.doi.org/10.1155/2014/638747). Lenvatinib also showed significant antitumor activity in all 5 ATC xenograft models at doses of 10 and 100 mg/kg (Figure 1(b), Supplementary Figure S1(b)). In addition, lenvatinib inhibited in vivo tumor growth of TT xenografts in a dose-dependent manner at 10, 30, and 100 mg/kg, causing tumor shrinkage at 100 mg/kg (Figure 1(c), Supplementary Figure S1(c)). These results demonstrate that lenvatinib shows the significant antitumor activity in a panel of 11 human thyroid cancer xenograft models with different histology of thyroid cancers.

### 3.2. Antiangiogenesis Activity of Lenvatinib in Human Thyroid Cancer Xenograft Models in Nude Mice

**Figure 2:**
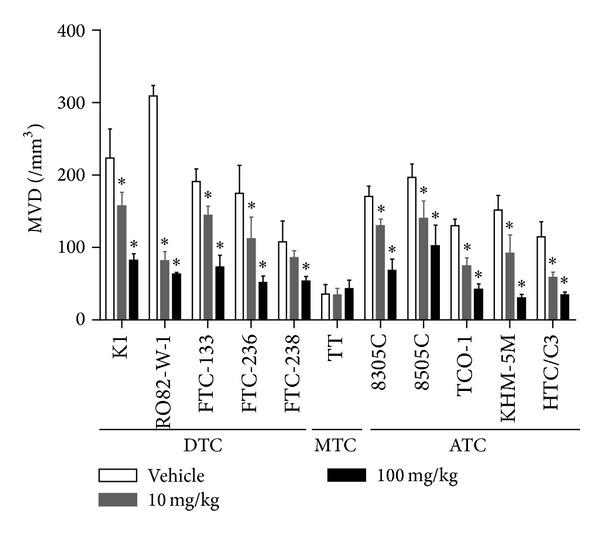
Antiangiogenesis activity of lenvatinib in human thyroid cancer xenograft models in nude mice. Nude mice bearing tumor xenografts were treated orally once daily with either vehicle or lenvatinib at the indicated doses when tumor volumes reached between 100 and 300 mm^3^ (day 1). Microvessel density (MVD) was analyzed by immunohistochemical staining of endothelial cells with an anti-mouse CD31 antibody within the resected tumor xenografts as described in Section 2. MVD is expressed as the average number of microvessels per mm^2^ in 5 regions of interest (ROIs). Each group consisted of 5 mice. Data are shown as means ± SD. *P < 0.05 compared with vehicle-treated mice. DTC: differentiated thyroid cancer, MTC: medullary thyroid cancer, and ATC: anaplastic thyroid cancer.

The antiangiogenesis activity of lenvatinib was evaluated by using the same 11 human thyroid cancer xenograft models that were examined for antitumor activity. For immunohistochemical analysis tumor tissues were resected next days after the last administration from nude mice bearing tumor xenografts that had been treated with lenvatinib at a dose of either 10 or 100 mg/kg. Tumor microvessels were stained immunohistochemically with an antibody against endothelial cell marker CD31, and MVD within tumor tissues was analyzed (Figure 2). MVD was significantly decreased in 4 of the 5 DTC xenografts treated with lenvatinib at 10 mg/kg and in all of the 5 DTC xenografts that received lenvatinib at 100 mg/kg. The MVDs in the human MTC TT xenografts were the lowest with vehicle treatments among the 11 human thyroid cancer xenograft models tested, and lenvatinib did not decrease the MVDs within the TT xenografts. The MVDs in all 5 ATC xenograft models decreased significantly with lenvatinib treatments at both 10 and 100 mg/kg. These results demonstrate that lenvatinib shows antiangiogenesis activity in almost all DTC and ATC models but not in MTC one. This antiangiogenesis activity may explain the mechanism of action of lenvatinib's antitumor activity observed in Figure 1. However, another mechanism may also contribute to the antitumor activity of lenvatinib against the human MTC TT model, because lenvatinib did not decrease the MVD in this model.

We performed immunohistochemical analyses in 5 DTC xenograft models by staining pericytes and endothelial cells with an anti-*α*SMA antibody and anti-CD31 antibody, respectively (Supplementary Figure S2), because treatment with a VEGF signal inhibitor was reported to increase the association of pericytes with endothelial cells (pericyte coverage), an event related to the acquisition of resistance [41]. The percentage of pericyte coverage in K1 and RO82-W1 cells was lower than that in the other 3 DTC cell types (Supplementary Figure S3). Interestingly, K1 and RO82-W-1 also have higher MVDs (>200/mm^3^) than the other 3 DTC cell lines (<200/mm^3^) among 5 DTC xenograft models (Figure 2). Lenvatinib showed significant antitumor activity in the K1 and RO82-W-1 models at the lower doses of between 1 and 10 mg/kg. These vascular parameters (low pericyte coverage and high MVD) together with the antitumor activity of lenvatinib suggest that the antiangiogenesis activity of lenvatinib is the mechanism of action behind the antitumor effects of lenvatinib in these models.

### 3.3. Antiproliferative Activity of Lenvatinib against Human Thyroid Cancer Cell Lines In Vitro

We examined the antiproliferative activity of lenvatinib against 11 human thyroid cancer cell lines to determine whether the RTK signaling pathway has a role in the in vitro proliferation of human thyroid cancer cell lines. Antiproliferative activity of lenvatinib was evaluated by using IC_50_ values and the ratios of the IC_50_ values of the thyroid cancer cell lines relative to that of Nthy-ori 3-1 cells (Table 1). Lenvatinib did not show the potent in vitro antiproliferative activity for 9 out of the 11 cell lines with IC_50_ values being greater than 10 *μ*M (Supplementary Figure S4). Lenvatinib did, however, show antiproliferative activity against the human DTC RO82-W-1 and MTC TT cell lines, with IC_50_ values of 3.8 and 0.078 *μ*M, respectively; moreover, it was selective against these two DTC cell lines compared with normal thyroid cells (*T*/*N* = 0.25 and 0.01, resp.). These data suggest that RTK signaling pathways may have roles in oncogenic proliferation of these two human thyroid cancer cells lines.

### 3.4. Antiproliferative Activity of Selective Receptor Tyrosine Kinase Inhibitors against Human DTC Cell Lines In Vitro

To investigate which RTK signaling pathways participate in the proliferation of RO82-W-1 cells, we tested the activity of selective RTK inhibitors [42–46] targeting VEGFR (sorafenib), FGFR (PD166866 and PD173074), KIT (imatinib), and PDGFR (Ki6783) against 5 DTC cell lines including RO82-W-1 (Table 2), because lenvatinib inhibited these RTKs at IC_50_ values of less than 100 nM (Supplementary Table S1) and RO82-W-1 cells express the mRNA of some of these RTKs (Supplementary Figure S5). The inhibitory activity of each RTK inhibitor varied against normal thyroid cells (Nthy-ori 3-1). We first determined the IC_50_ values against thyroid cancer cell lines and Nthy-ori 3-1, and then, we compared the ratio of the IC_50_ values between the tumor cells and the normal cells (i.e., *T*/*N*). Among the 5 DTC cell lines, the RO82-W-1 cell line showed selective sensitivity (*T*/*N* ≤ 0.5) to FGFR inhibitors (*T*/*N* = 0.14 for both PD166866 and PD173074). No cell lines showed selective sensitivity to any of the RTK inhibitors we examined except for PD166866, PD173074, and lenvatinib (*T*/*N* = 0.26) against RO82-W-1 cells; thus, lenvatinib might target the FGFR signaling pathway to inhibit the proliferation of RO82-W-1 cells.

### 3.5. Effect of the Selective FGFR Kinase Inhibitor PD173074 on the FGFR Signaling Pathway in Human DTC RO82-W-1 Cells

**Figure 3:**
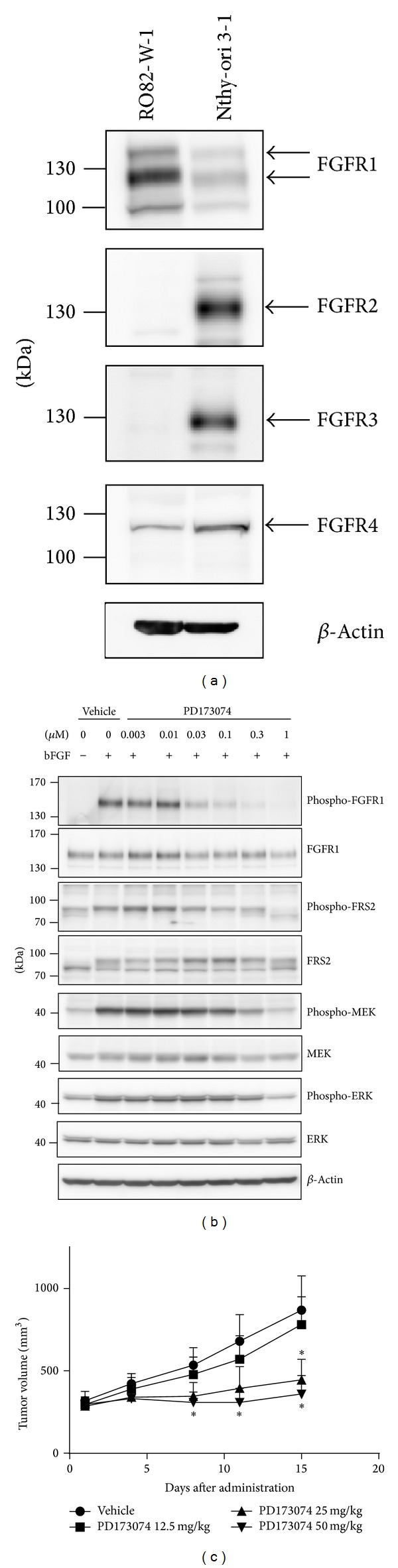
Effect of the selective FGFR kinase inhibitor PD173074 on the FGFR signaling pathway in human DTC RO82-W-1 cells. (a) Western blot analysis of the expression of FGF receptors in vitro. Expression levels of FGF receptors in RO82-W-1 cells were compared to those in Nthy-ori 3-1 cells (normal thyroid cells). (b) Western blot analysis of the effects of PD173074 on the phosphorylation of FGFR1 and its downstream effectors. After starvation overnight, RO82-W-1 cells were treated with PD173074 at the indicated concentrations for 1 h and were then stimulated for 10 min with bFGF (20 ng/mL) and heparin before being lysed. (c) Antitumor activity of PD173074 against RO82-W-1 xenografts in nude mice. Nude mice bearing RO82-W-1 xenografts were treated orally once daily for 14 days with either vehicle or PD173074 at the indicated doses when tumor volumes reached about 300 mm^3^ (day 1). The tumor volume was measured on the indicated days after administrations. Each group consisted of 5 mice. Data are shown as means ± SD. *P < 0.05 compared with vehicle-treated mice.

Our data suggested a role of the FGFR signaling pathway in the in vitro proliferation of RO82-W-1 cells. Therefore, we performed western blot analysis to examine whether the expression levels of FGFR1–4 were upregulated in RO82-W-1 cells compared with normal thyroid cells (Figure 3(a)), because quantitative RT-PCR suggested overexpression of some of FGFRs (Supplementary Figure S5). Among the 4 FGFRs, the expression of FGFR1 was upregulated in RO82-W-1 cells compared with that in normal thyroid cells, whereas FGFR2 and FGFR3 protein expression was downregulated. FGFR4 protein was expressed at similar levels in RO82-W-1 and normal thyroid cells. Next, we examined whether PD173074, a selective FGFR kinase inhibitor, affected FGFR1-mediated signaling pathways in RO82-W-1 cells in vitro by performing western blot analysis to evaluate the phosphorylation status of FGFR1 and its downstream effectors (i.e., FRS2, MEK, and ERK) (Figure 3(b)). PD173074 inhibited the bFGF-induced phosphorylation of FGFR1, FRS2, MEK, and ERK in a concentration-dependent manner, suggesting that the FGFR1 signaling pathway is active in RO82-W-1 cell lines. To evaluate the role of FGFR signaling in the tumorigenesis of the RO82-W-1 cell line, we examined the antitumor effect of PD173074 against the in vivo tumor growth of RO82-W-1 xenografts in nude mice (Figure 3(c)). Daily oral administration of PD173074 for 14 days led to significant antitumor activity in the RO82-W-1 xenograft models. These results indicate that the FGFR1 signaling pathway participates in the tumorigenesis of the human DTC RO82-W-1 cell line.

### 3.6. Effect of Lenvatinib on FGFR1 Signaling Pathway in the Human DTC RO82-W-1 Model

**Figure 4:**
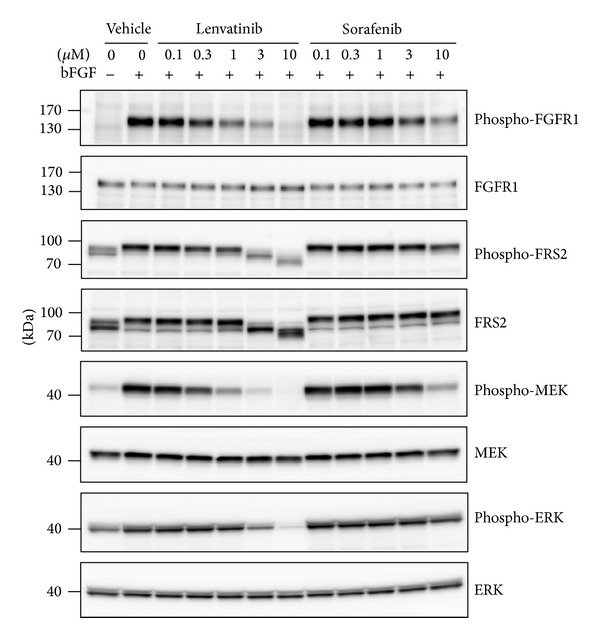
Effect of lenvatinib on FGFR1 signaling pathway in human DTC RO82-W-1 cells in vitro. After starvation overnight, RO82-W-1 cells were treated with vehicle (control), lenvatinib or sorafenib at the indicated concentrations for 1 h and were then stimulated for 10 min with bFGF (20 ng/mL) and heparin before being lysed. Western blot analyses of the phosphorylation of FGFR1 and its downstream effectors in RO82-W-1 cells were then performed and representative images were shown.

We examined whether lenvatinib inhibited FGFR1 signaling pathways in RO82-W-1 cells in vitro by performing western blot analysis to evaluate the phosphorylation status of FGFR1 and its downstream effectors (Figure 4). Lenvatinib inhibited the phosphorylation of FGFR1 and of FRS2, MEK, and ERK, in a concentration-dependent manner at a similar concentration to that at which it showed antiproliferative activity (3 *μ*M). Lenvatinib-induced inhibition of phosphorylation of FRS2 was also detected as a band-shift of FRS2 in vitro. We also examined whether sorafenib affected the FGFR1 signaling pathway in WO82-W-1 cells as a reference. Sorafenib, another multitargeted kinase inhibitor, targets KIT, FLT-3, RAF1, RET, VEGFR1–3, and PDGFR*β* but is not reported to inhibit the FGFR signaling pathway. Sorafenib weakly inhibited phosphorylation of MEK at 10 *μ*M but did not clearly inhibit the phosphorylation of FGFR1 or its other downstream effectors, even though the concentration used was sufficient to produce antiproliferative effects in RO82-W-1 cells, with an IC_50_ value 4.2 *μ*M (Table 2). We also tested whether lenvatinib inhibited the phosphorylation of FRS2 in RO82-W-1 xenografts in nude mice (Supplementary Figure S6). Two hours after administration of lenvatinib at 3, 10, 30, or 100 mg/kg, the phosphorylation of FRS2 was decreased in RO82-W-1 xenografts. However, we could not detect a clear decrease in the phosphorylation of FRS2 along with band shift with sorafenib treatment at 100 mg/kg. These data demonstrate that lenvatinib inhibits the FGFR signaling pathway both in vitro and in vivo and suggest that the antitumor effect of lenvatinib against RO82-W-1 xenografts is attenuated through the inhibition of FGFR signaling in cancer cells, besides antiangiogenic activity through the inhibition of VEGFR2 signaling in endothelial cells. We also examined the activity of sorafenib in the RO82-W-1 DTC model both in vitro and in vivo. Sorafenib did not inhibit the phosphorylation of FGFR1 in RO82-W-1 cells in vitro even at concentrations that produced antiproliferative activity (Table 2 and Figure 4). Orally administered sorafenib inhibited tumor growth of RO82-W-1 xenografts in nude mice at doses between 30 and 300 mg/kg (Supplementary Figure S7). At the highest dose of lenvatinib (100 mg/kg) compared with that of sorafenib (300 mg/kg), lenvatinib showed significant potent antitumor activity against tumor growth of RO82-W-1 xenografts in nude mice (Supplementary Figure S7). When we compared antiangiogenesis activity with antitumor activity for each treatment, we found that lenvatinib showed stronger antitumor activity (percentage of tumor growth inhibition, %TGI) than did sorafenib when the two treatments were normalized for angiogenesis inhibition as determined by decreases in MVD (percentage of microvessel inhibition, %MVI) (Supplementary Figure S7). These results suggest that some additional activity of lenvatinib, besides its effect on antiangiogenesis, may contribute to its more potent antitumor activity in RO82-W-1 xenograft models.

### 3.7. Effects of Lenvatinib on the Activated RET Signaling Pathway in Human MTC TT Cells

**Figure 5:**
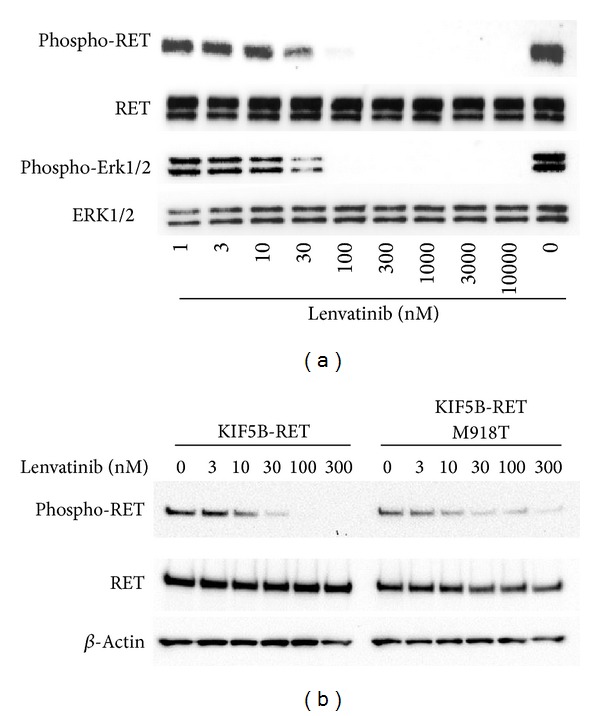
Effect of lenvatinib on the phosphorylation of RET with an activating mutation or a rearrangement in vitro. (a) Western blot analyses of the phosphorylation of RET in human medullary thyroid TT cells. TT cells were seeded and cultured overnight. They were then treated with lenvatinib at the indicated concentrations for 1 h before being lysed. (b) Western blot analyses of the phosphorylation of KIF5B-RET fusion proteins in normal thyroid cells; Nthy-ori 3-1 transfectants overexpressing KIF5B-RET (wild-type) or KIF5B-RET (M918T). Nthy-ori 3-1 transfectants were cultured overnight and then treated with lenvatinib at the indicated concentrations for 1 h before being lysed. Western blot analyses of the phosphorylation of RET and its downstream effectors in Nthy-ori 3-1 transfectants were then performed and representative images were shown.

Lenvatinib showed potent antiproliferative activity against human MTC TT cells with an IC_50_ value of 0.078 *μ*M in vitro (Table 1). Because TT cells carry the C634W activating mutation of RET [47], lenvatinib might exert an antiproliferative effect on TT cells by inhibiting the RET RTK. To examine whether lenvatinib affects the RET signaling pathway that is activated by C634W mutation we performed western blot analysis to detect phosphorylation of RET and its downstream effector phosphor-ERK1/2 in vitro. Treatments with lenvatinib for 1 h inhibited the phosphorylation of RET and of ERK1/2 in cultured TT cells (Figure 5(a)). In addition, inhibition of RET phosphorylation in TT xenografts was also observed 2 h after oral administrations of lenvatinib (Supplementary Figure S8) at doses that produced antitumor activity as shown in Figure 1(c). We also examined the effect of lenvatinib on the phosphorylation of RET with the M918T mutant. We generated transfectants overexpressing the fusion genes of KIF-RET (wild-type) and KIF-RET (M918T) in Nthy-ori 3-1 cells to compare the effect of lenvatinib on phosphorylation of wild-type RET and M918T mutant. Lenvatinib inhibited the phosphorylation of both wild-type RET and M918T mutant (Figure 5(b)) at a similar concentration to that which inhibited the phosphorylation of RET (C634W mutant). These data demonstrate that lenvatinib inhibits the RET signaling pathway that is activated by mutations in RET in vitro and lenvatinib thus may show significant antitumor effects in the human MTC TT xenograft model.

### 3.8. Molecular Mutation Profile of Human DTC Cell Lines

We determined the gene mutation profiles of 5 DTC cell lines by using the MassARRAY System to evaluate 443 mutations in 32 genes. A summary of our mutation analysis is shown in Supplementary Table S2. K1 cells were found to carry the BRAF mutation (V600E) and the PIK3CA mutation (E542K). FTC-133, FTC-236, and FTC-238 cells have a PTEN deletion mutation (R130*) and the TP53 mutation (R273H). We did not detect any RAS mutations among the 5 DTC cell lines.

## 4. Discussion

In this report, we determined the antitumor and antiangiogenesis activities of lenvatinib, an angiogenesis inhibitor that targets multiple RTKs, in 11 human thyroid cancer xenograft models with 3 types of histology (DTC, MTC, and ATC). Orally administered lenvatinib significantly inhibited tumor growth of 1 PTC (a major type of DTC), 4 FTC (another major type of DTC), 1 MTC, and 5 ATC xenografts in nude mice. Lenvatinib inhibited tumor angiogenesis in 5 DTC and 5 ATC xenograft models as evidenced by a decrease in MVD. Our finding that lenvatinib did not show potent antiproliferative activity against 9 out of 11 human thyroid cancer cell lines in vitro suggests the antitumor activity of lenvatinib against a broad panel of human thyroid cancer models primarily from its antiangiogenic effects.

Lenvatinib inhibited the in vitro proliferation of some of the thyroid cancer cell lines: that is, the RO82-W-1 and TT cell lines. We performed qRT-PCR analysis to examine the mRNA expression levels of 13 RTKs including lenvatinib-targeted RTKs, in 5 DTC cell lines compared with those in normal thyroid cells (Supplementary Figure S5). FGFR1 mRNA levels were upregulated in WO82-W-1 cells compared to Nthy-ori 3-1 and expressed the highest level of FGFR1 mRNA. Both KIT and PDGF*α* mRNA were highly expressed in RO82-W-1 cells. However, selective RTK inhibitors of KIT (imatinib) and PDGFR*α* (Ki6783) showed no antiproliferative activity against RO82-W-1, but FGFR inhibitors (PD166866 and PD174073) did. Consistent with these data, western blot analysis showed that RO82-W-1 cells overexpress FGFR1 protein. Together these results suggest a selective role of FGFR signaling pathway in the RO82-W-1 cell line.

Our results showed that lenvatinib inhibited the FGFR signaling pathway in vitro and in vivo (Figure 4 and Supplementary Figure S6). Although lenvatinib showed the inhibitory activity against the VEGFR1–R3, FGFR1–R4, KIT, RET, and PDGFR*α* tyrosine kinases (Supplementary Table S1), our data suggest that lenvatinib showed direct antitumor activity against RO82-W-1 cell lines by inhibiting the FGFR signaling pathway. FGFR1 and FGFR3 are expressed in well-differentiated thyroid cancers, and ATC cells overexpress FGFR4 [35]. It will be interesting to investigate whether lenvatinib shows antitumor activity against human thyroid cancers with active FGFR signaling pathways.

We found that lenvatinib showed potent antiproliferative activity in vitro (IC_50_ value of 0.078 *μ*M) against TT MTC cells, in which the RET signaling pathway was activated by a mutation in RET (C634W). As a reference, sorafenib showed antiproliferative activity against TT cells with an IC_50_ value of 0.26 *μ*M. Lenvatinib inhibited the phosphorylation of RET both in vitro (Figure 5) and in vivo (Supplementary Figure S8). We did not detect a significant decrease in MVD in the TT xenograft models, although lenvatinib showed clear antitumor activity, causing tumor shrinkage at 100 mg/kg (Figure 1). Therefore, the antiproliferative activity of lenvatinib may contribute to its anticancer activity in TT MTC xenograft models. We also examined the effect of lenvatinib on the phosphorylation of RET carrying another activated mutation (M918T) in Nthy-ori 3-1 cells that ectopically expressed either KIF5B-RET (wild-type) or KIF5B-RET (M918T) (Figure 5(b)). Lenvatinib inhibited both the wild-type and M918T mutant RET proteins at similar concentrations in vitro. We previously reported that lenvatinib inhibits the phosphorylation of CCDC-RET and both the in vitro proliferation and in vivo tumor growth of human DTC TPC1 cell lines [37]. These results demonstrate that lenvatinib inhibits RET signaling in the presence of genetic alterations in RET such as an activated mutation or gene rearrangement. In a phase 3 trial in MTC patients, vandetanib, a VEGFR2/RET inhibitor, showed clinical benefits that were independent of RET gene mutation status [34], suggesting that multiple receptor tyrosine kinase inhibitors may be able to show antitumor activity based on antiangiogenesis activity targeting VEGFR2. In the TT xenograft model, we did not detect a clear decrease in MVD. Lenvatinib showed significant antitumor activity against this model with inhibition of the phosphorylation of RET, suggesting that the antitumor activity of lenvatinib against TT cells was derived from RET inhibition. However, further investigation is needed to determine whether the antiangiogenesis activity of lenvatinib participates in its antitumor activity against MTCs with activated mutations in RET.

Molecular profiling of the 5 DTC cell lines showed that K1 cells carry the BRAF V600E mutation (Supplementary Table S2). Activation of the MAPK pathway due to gene mutations in RAS and BRAF has been associated with malignant phenotypes in thyroid cancer [48]. Because lenvatinib showed antitumor and antiangiogenesis activity in the K1 DTC xenograft model at low doses (between 1 and 10 mg/kg), the activated BRAF mutation might not be involved in resistance to antiangiogenesis therapy in this preclinical model. Taken together, these results suggest that lenvatinib has antitumor activity despite the presence of genetic alterations related to thyroid cancer in the 11 preclinical thyroid cancer models we examined.

In conclusion, lenvatinib showed promising antitumor activity in 11 human thyroid cancer xenograft models derived from DTC, MTC, and ATC cell lines. Lenvatinib showed antitumor activity in most of the human thyroid cancer models tested due to its antiangiogenesis activity. In addition, the activity of lenvatinib through the inhibition of multiple RTKs may contribute to its antitumor activity against thyroid cancer cell lines with gene alterations (e.g., mutations or rearrangements) and overexpression of targeted RTKs, such as RET and FGFR1. Lenvatinib is thus an effective therapeutic agent in most thyroid cancer xenograft models.

## Supplementary Material

The Supplementary Material has contained additional 2 tables and 8 figures: Supplementary Table S1 Kinase inhibition profile of lenvatinib. Supplementary Table S2 Molecular Profile of human differentiated thyroid cancer cell lines. Supplementary Fig. S1 Tumor growth curves in human thyroid cancer xenograft models. Supplementary Fig. S2 Immunohistochemical analysis of tumor microvessels in human differentiated thyroid cancer xenograft models. Supplementary Fig. S3 Immunohistochemical analysis of pericyte coverage in human differentiated thyroid cancer xenograft models. Supplementary Fig. S4 Antiproliferative activity of lenvatinib against human thyroid cancer cells in vitro. Supplementary Fig. S5 Quantitative PCR analysis of receptor tyrosine kinase mRNA expression in human differentiated thyroid cancer cell lines. Supplementary Fig. S6 Effect of lenvatinib on the phosphorylation of FRS2 in human differentiated thyroid cancer RO82-W-1 xenografts. Supplementary Fig. S7 Comparison of antitumor activity with antiangiogenesis activity of lenvatinib and sorafenib in the human differentiated thyroid cancer RO82-W-1 xenograft model. Supplementary Fig. S8 Effect of lenvatinib on the phosphorylation of RET in human medullary thyroid cancer TT xenografts.

## Figures and Tables

**Table 1:** In vitro antiproliferative activity of lenvatinib against human thyroid cancer cells.

Tissue type	Cell line	Lenvatinib	
		IC_50_^1^ (*μ*M)	T/N^2^
Normal	Nthy-ori 3-1	15	—

DTC^3^	K1	22	1.47
	RO82-W-1	3.8	0.25
	FTC-133	24	1.6
	FTC-236	17	1.13
	FTC-238	18	1.2

MTC^4^	TT	0.078	0.01

ATC^5^	8305C	26	1.73
	8505C	26	1.73
	TCO-1	28	1.87
	KHM-5M	28	1.87
	HTC/C3	28	2.53

^1^The half maximal inhibitory concentration.

^2^T/N: ratio of IC_50_ values of thyroid cancer cell lines to that of Nthy-ori 3-1 cells.

^3^DTC: differentiated thyroid cancer.

^4^MTC: medullary thyroid cancer.

^5^ATC: anaplastic thyroid cancer.

**Table 2:** In vitro antiproliferative activity of selective receptor tyrosine kinase inhibitors against human DTC cells.

Tissue type	Cell line	VEGFR2i^1^		FGFRi		FGFRi		KITi		PDGFi	
		Sorafenib		PD166866		PD173074		Glivec		Ki6783	
		IC_50_^3^ (*μ*M)	T/N^4^	IC_50_^3^ (*μ*M)	T/N^4^	IC_50_^3^ (*μ*M)	T/N^4^	IC_50_^3^ (*μ*M)	T/N^4^	IC_50_^3^ (*μ*M)	T/N^4^
Normal	Nthy-ori 3-1	5.7	—	14	—	4.6	—	20	—	1.7	—

DTC^2^	K1	5.2	0.91	29	2.07	11	2.39	21	1.1	9.2	5.4
	RO82-W-1	4.2	0.73	1.9	0.14	0.63	0.14	21	1.1	2.5	1.5
	FTC-133	5.5	0.96	23	1.64	9.7	2.11	23	1.2	8	4.7
	FTC-236	7.7	1.35	30<	2.14<	4.3	0.93	30<	2.14<	30<	2.14<
	FTC-238	7.3	1.28	21	1.5	9.4	2.04	24	1.3	2	1.2

^1^Inhibitor.

^2^Differentiated thyroid cancer.

^3^IC_50_: the half maximal inhibitory concentration.

^4^T/N ratio of IC_50_ values of thyroid cancer cell lines to that of Nthy-ori 3-1 cells.
