# Supplementary material for: Antitumor Activity of Lenvatinib (E7080): An Angiogenesis Inhibitor That Targets Multiple Receptor Tyrosine Kinases in Preclinical Human Thyroid Cancer Models
Source: J Thyroid Res. 2014 Sep 10;2014:638747. doi: 10.1155/2014/638747 (PMC4177084; doi:10.1155/2014/638747)
Supplement: Supplementary file 1 — The Supplementary Material has contained additional 2 tables and 8 figures: Supplementary Table S1 Kinase inhibition profile of lenvatinib. Supplementary Table S2 Molecular Profile of human differentiated thyroid cancer cell lines. Supplementary Fig. S1 Tumor growth curves in human thyroid cancer xenograft models. Supplementary Fig. S2 Immunohistochemical analysis of tumor microvessels in human differentiated thyroid cancer xenograft models. Supplementary Fig. S3 Immunohistochemical analysis of pericyte coverage in human differentiated thyroid cancer xenograft models. Supplementary Fig. S4 Antiproliferative activity of lenvatinib against human thyroid cancer cells in vitro. Supplementary Fig. S5 Quantitative PCR analysis of receptor tyrosine kinase mRNA expression in human differentiated thyroid cancer cell lines. Supplementary Fig. S6 Effect of lenvatinib on the phosphorylation of FRS2 in human differentiated thyroid cancer RO82-W-1 xenografts. Supplementary Fig. S7 Comparison of antitumor activity with antiangiogenesis activity of lenvatinib and sorafenib in the human differentiated thyroid cancer RO82-W-1 xenograft model. Supplementary Fig. S8 Effect of lenvatinib on the phosphorylation of RET in human medullary thyroid cancer TT xenografts. [file 638747.f1.pdf]

Supplementary Table S1 Kinase inhibition profile in 66 kinases

	Lenvatinib	Sorafenib
Kinase	IC_{50}[1)] (μM)	IC_{50}[1)] (μM)
KIT(V560G)	0.00074	0.0046
FLT4/VEGFR3	0.0023	0.016
KDR/VEGFR2	0.0030	0.021
FLT1/VEGFR1	0.0047	0.021
RET	0.0064	0.015
RET(M918T)	0.012	0.033
PDGFRα(V561D)	0.025	0.0054
FGFR2	0.027	0.15
PDGFRα	0.029	0.0016
FGFR4	0.043	3.4
FGFR3	0.052	0.34
FGFR1	0.061	0.34
KIT	0.085	0.14
FGFR3(K650E)	0.11	0.10
LCK	0.13	0.96
FRK	0.16	0.37
PDGFRβ	0.16	0.027
HER4	0.17	>10
BRK	0.17	8.3
FGFR3(K650M)	0.25	0.11
HGK	0.40	3.3
KIT(T670I)	0.41	0.060
KIT(V654A)	0.52	0.92
MET	0.52	>10
EGFR	0.62	>10
PDGFRα(T674I)	0.63	0.086
ABL	0.66	1.1
EPHB2	0.66	0.71
FGR	0.91	1.2
AurA	1.1	2.5
RAF1	1.6	0.046
TIE2	2.5	1.7
CSK	2.6	5.3

	Lenvatinib	Sorafenib
Kinase	IC_{50}[1)] (μM)	IC_{50}[1)] (μM)
FLT3	4.1	0.057
EPHB4	4.6	0.66
EPHA1	5.1	1.2
SRC	5.7	4.5
BRAF	8.7	0.31
SYK	8.7	>10
EPHB3	>10	>10
FAK	>10	>10
HER2	>10	>10
IGF1R	>10	>10
INSR	>10	>10
JAK2	>10	4.4
KIT(D816V)	>10	1.4
MUSK	>10	0.068
AKT1	>10	>10
BRAF(V600E)	>10	0.38
CDK2/CycE1	>10	>10
CDK4	>10	>10
CDK6	>10	>10
CHK1	>10	>10
COT	>10	>10
GSK3β	>10	>10
IKKb	>10	>10
IRAK4	>10	>10
MLK1	>10	>10
NEK2	>10	>10
p70S6K	>10	0.46
PIM1	>10	>10
PKACα	>10	>10
PLK1	>10	>10
PRKX	>10	>10
SGK3	>10	>10
WEE1	>10	>10

[1)] IC_{50}: The half maximal inhibitory concentration

Supplementary Table S2 Molecular profile of human differentiated thyroid cancer cell lines

Cell line	Mutation					
	PTEN	RB1	TP53	BRAF	PIK3CA	JAK3
K1				V600E	E542K	
RO82-W-1						V722I
FTC-133	R130*		R273H			
FTC-236	R130*	R320	R273H			
FTC-238	R130*	R320	R273H			

* stop codon

Gene mutation analysis Cells (2 × 10^6) were seeded and cultured in 6-well culture plates. After overnight culture, genomic DNA was isolated from the cultured cells (K1, RO82-W-1, FTC-133, FTC-236, and FTC-238) by using the DNeasy Blood & Tissue kit (Qiagen, Hilden, Germany). Mutation analysis for 443 mutations among 32 genes mutation [*ABL1* (16), *AKT1* (8), *AKT2* (2), *APC* (12), *BRAF* (44), *CDK4* (2), *CDKN2A* (7), *CSF1R* (6), *CTNNB1* (28), *EGFR* (72), *FGFR1* (2), *FGFR3* (7), *FLT3* (6), *HRAS* (12), *JAK2* (1), *JAK3* (3), *KIT* (35), *KRAS* (21), *MET* (11), *MLH1* (1), *NRAS* (19), *P53* (7), *PDGFRA* (22), *PIK3CA* (18), *PTEN* (14), *RB1* (11), *RET* (19), *SRC* (1), *STK11* (12), *VH1* (7)] was performed by using the MassARRAY System (Sequenom, San Diego, CA) with OncoCarta Panel versions 1.0 and 3.0.

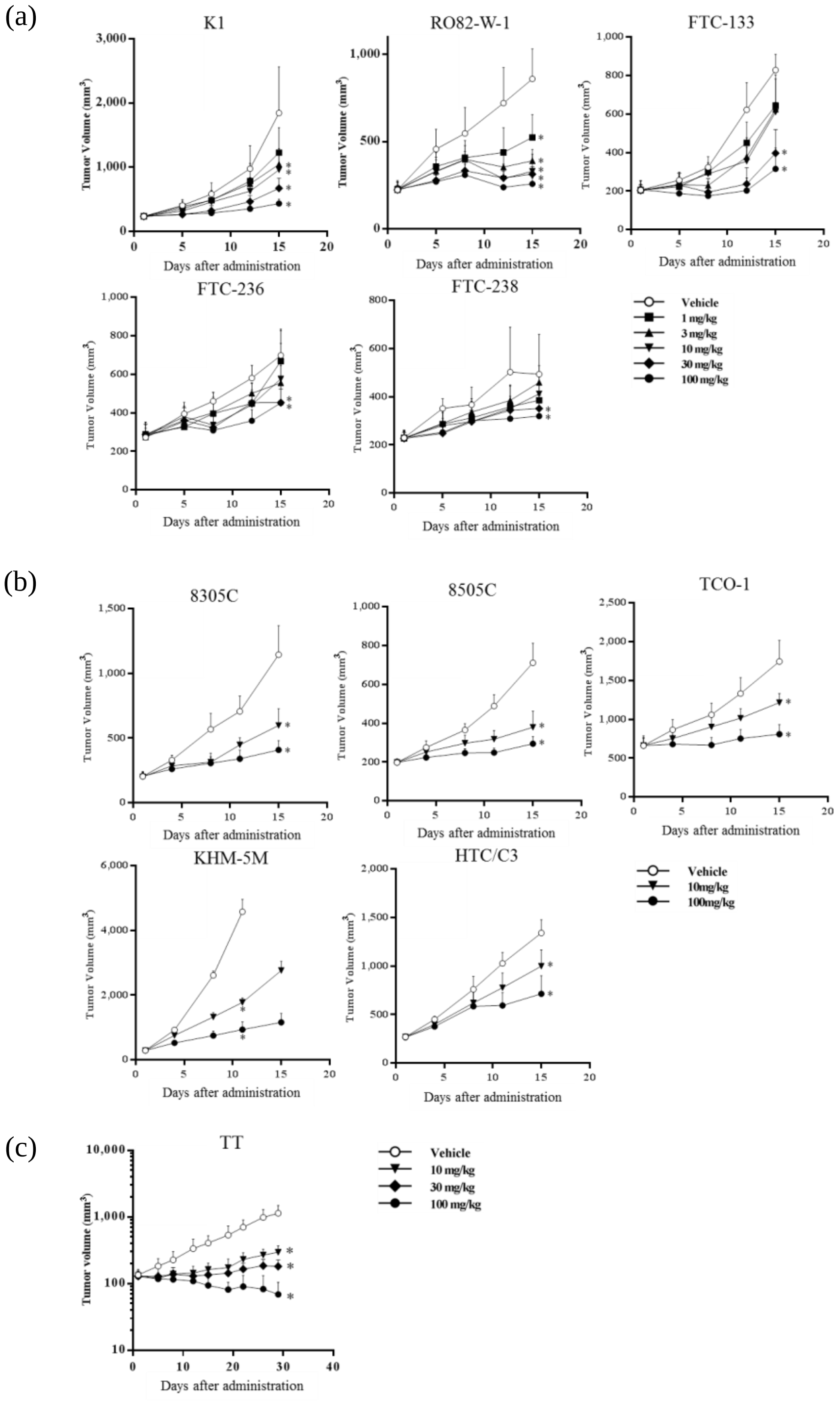

Supplementary Fig. S1 Tumor growth curves of human thyroid cancer xenografts in nude mice. Nude mice bearing tumor xenografts were treated orally once daily with either vehicle or lenvatinib at the indicated doses when tumor volumes reached between 100 and 300 mm^3 (day 1). Each group consisted of 5 mice. (a) DTC xenograft models. (b) ATC xenograft models. (c) MTC (TT) xenograft models. Tumor volumes are shown for the indicated days. Data are shown as means ± SD. * $P < 0.05$ compared with vehicle-treated mice at the end of each experiment.

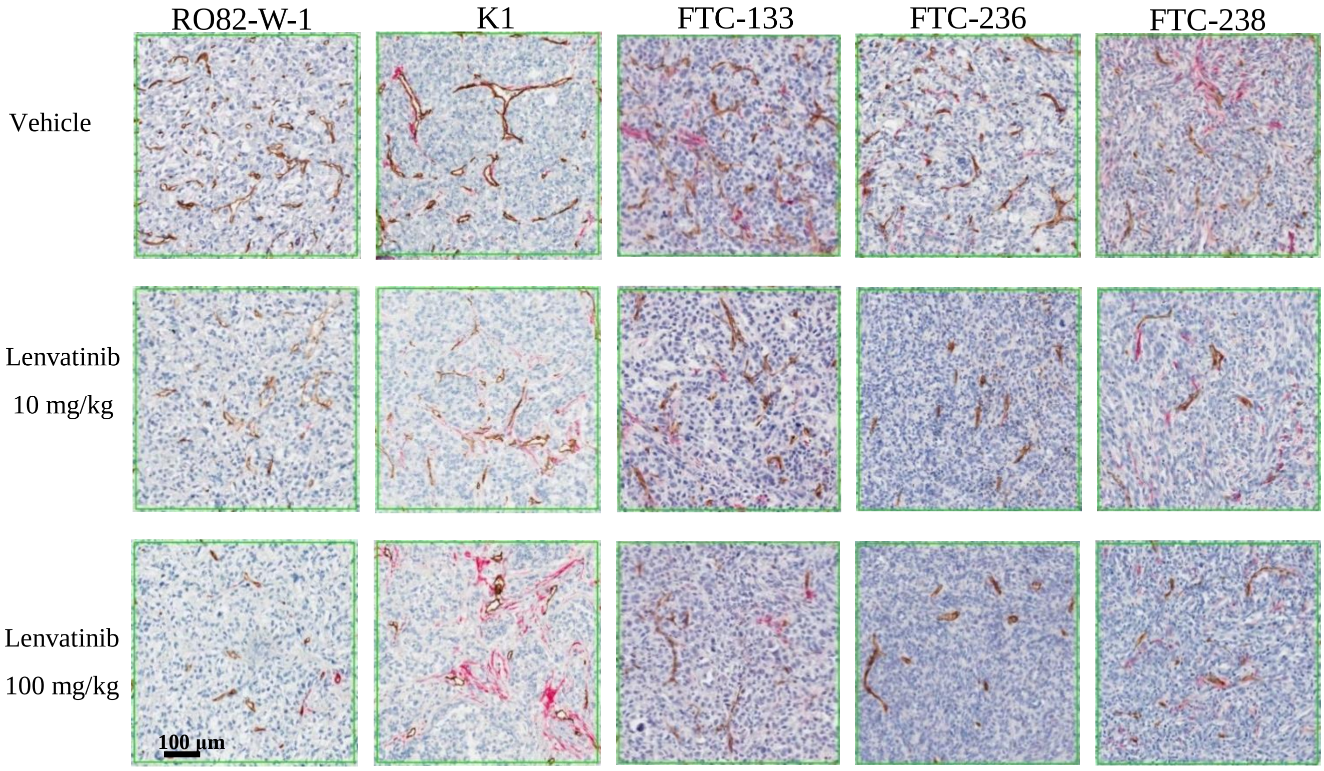

Supplementary Fig. S2 Immunohistochemical analysis of tumor microvessels in human differentiated thyroid cancer xenograft models. Nude mice bearing tumor xenografts were treated orally once daily for 14 days with either vehicle or lenvatinib at the indicated doses, when tumor volumes reached approximately 100-300 mm^3 (Figure 1(a)). Tumor tissues were resected from the nude mice next days after the last administration, and immunohistochemical analysis was performed. Tumor microvessels were analyzed by means of immunohistochemical analysis of endothelial cells stained with an anti-mouse CD31 antibody (brown; endothelial cells) and an anti-αSMA antibody (red; pericytes) within resected tumor xenografts as described in the Materials and Methods. Representative images from each group are shown.

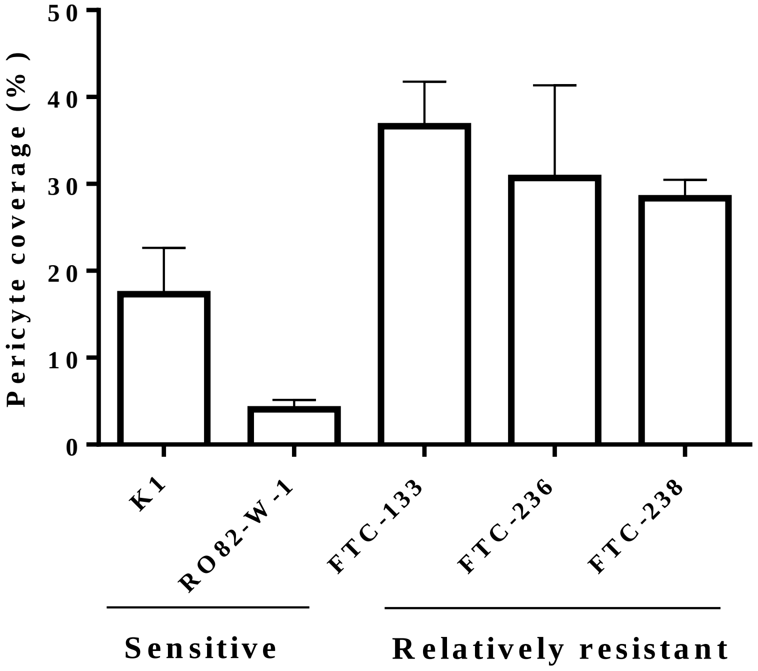

Supplementary Fig. S3 Immunohistochemical analysis of pericyte coverage in human differentiated thyroid cancer (DTC) xenograft models in nude mice. Tumor microvessels were analyzed by immunohistochemical staining with an anti-mouse CD31 antibody (endothelial cells) and an anti-αSMA antibody (pericytes) as described in the Materials and Methods. The percentage of pericyte-covered microvessels (pericyte coverage) is performed based on the same DTC models in Figure 2 and is shown as the number of pericyte-covered microvessels / total number of microvessels × 100 in the selected area. Each group consisted of 5 mice. Sensitive: lenvatinib at 1 to 100 mg/kg showed significant in vivo antitumor activity. Relatively resistant: lenvatinib showed significant in vivo antitumor activity at 30 to 100 mg/kg. Data are shown as means ± SD.

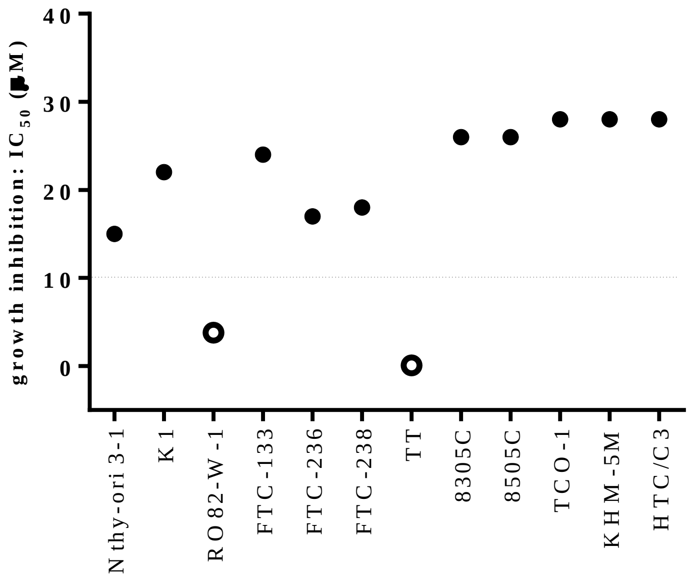

Supplementary Fig. S4 Antiproliferative activity of lenvatinib against human thyroid cancer cells in vitro. IC_{50} (the half maximal inhibitory concentration) values of each cell line in Table 1 were shown.

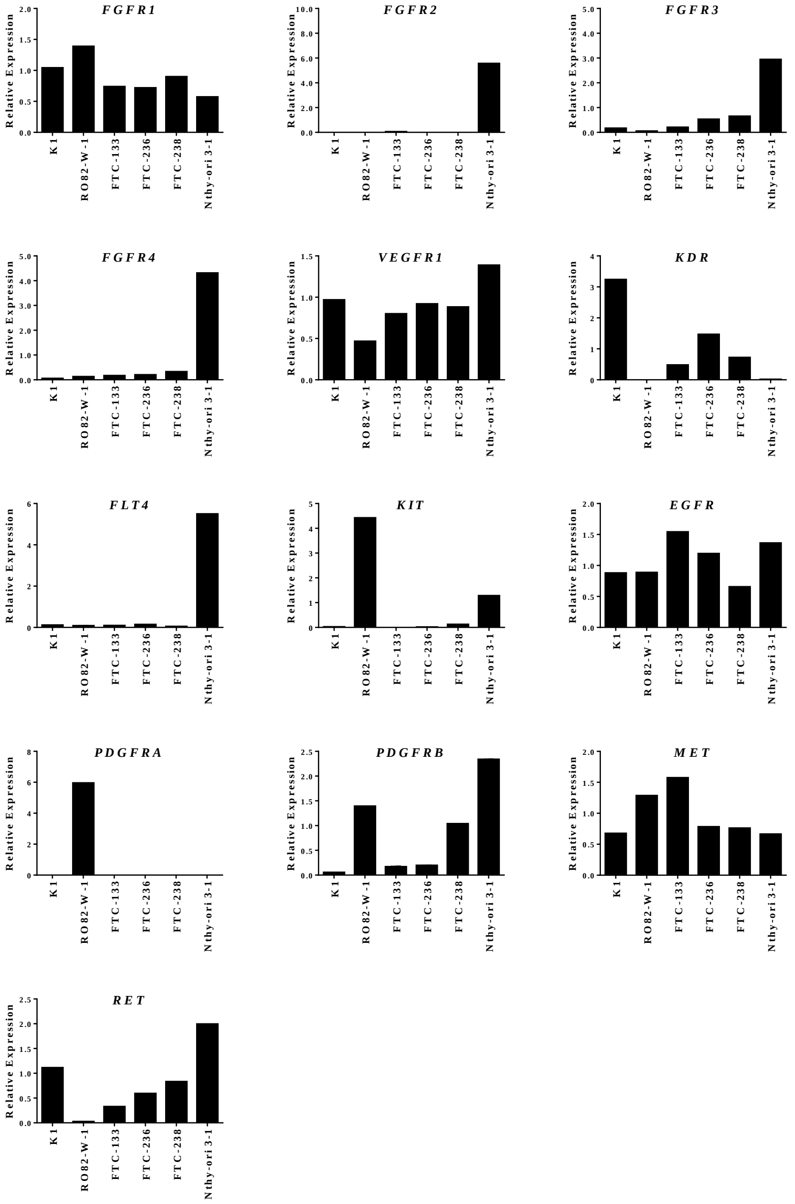

Supplementary Fig. S5 Quantitative PCR analysis of receptor tyrosine kinase mRNA expression in human differentiated thyroid cancer (DTC) cell lines. DTC cell lines and normal thyroid Nthy-ori 3-1 cells were seeded (1×10^5 cells) in 6-well culture plates and cultured overnight. Total RNA was then isolated from the cultured cells by using an RNeasy Mini Kit (Qiagen, Hilden, Germany). cDNA was synthesized from total RNA using a High Capacity cDNA Reverse Transcription Kit (Life Technologies, Carlsbad, CA). Synthesized cDNA was used as a template for quantitative polymerase chain reaction (PCR) assays using TaqMan Universal PCR Master Mix (Life Technologies), AmpErase UNG (Life Technologies) and analyzed by the real-time PCR method using the TaqMan probes as described in the Materials and Methods. Cycle threshold (Ct) values were determined by using SDS software (Life Technologies). Experiments were performed in duplicate and the mean relative gene expression for each gene (relative to *18S rRNA*) is shown.

(a)

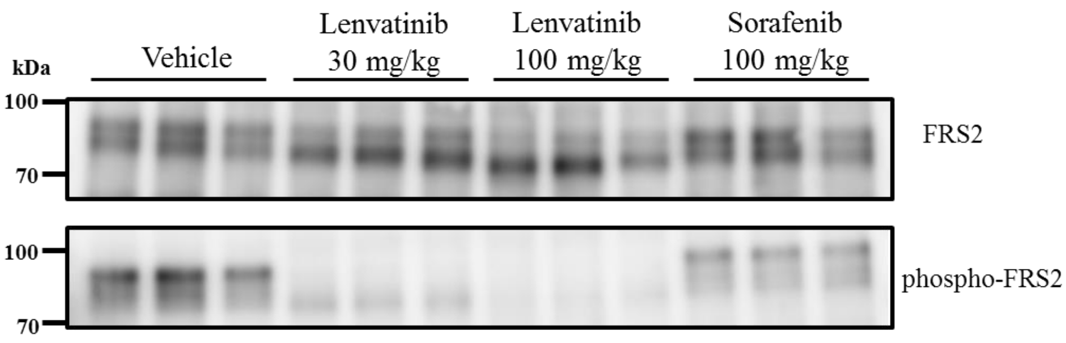

(b)

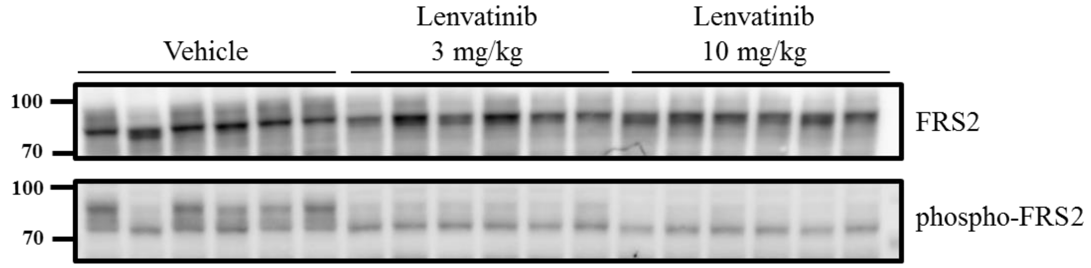

Supplementary Fig. S6 Effect of lenvatinib on the phosphorylation of FRS2 in human differentiated thyroid cancer RO82-W-1 cells in nude mice. Western blot analysis of the phosphorylation of FRS2 in human RO82-W-1 xenografts was performed. Nude mice bearing tumor xenografts were treated once orally with vehicle, lenvatinib, or sorafenib at the indicated doses (n = 3 or 6), when tumor volumes reached approximately 100-300 mm^3. Tumors were collected 2 h after administration, lysed with RIPA buffer, and used for western blot analysis as described in the Materials and Methods. (a) Lenvatinib (30 and 100 mg/kg) and sorafenib (100 mg/kg), (b) Lenvatinib (3 and 10 mg/kg).

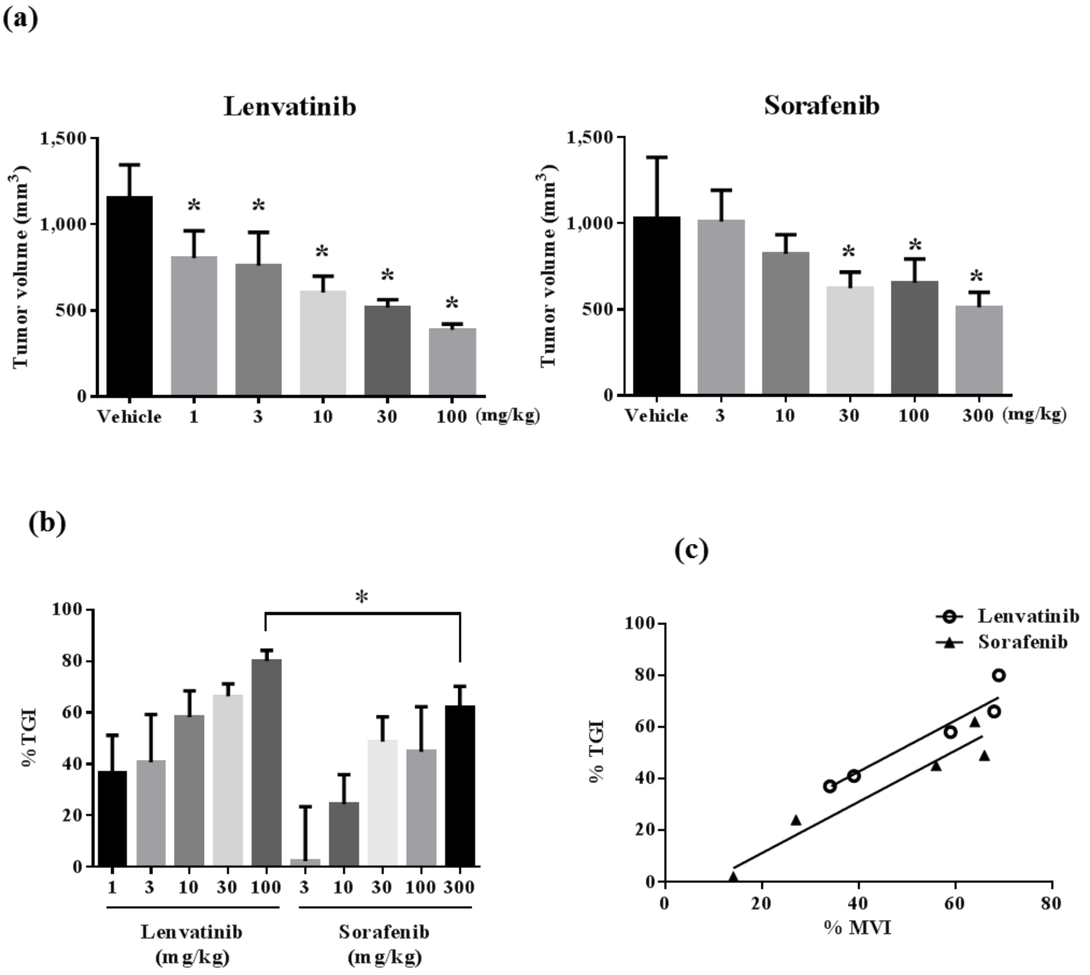

Supplementary Fig. S7 Comparison of antitumor activity with antiangiogenesis activity in human differentiated thyroid cancer RO82-W-1 xenografts in nude mice. (a) Antitumor activity of lenvatinib and sorafenib against RO82-W-1 xenografts in nude mice. Nude mice bearing RO82-W-1 xenografts were treated orally once daily for 21 days with vehicle, lenvatinib and sorafenib at the indicated doses when tumor volumes reached about 200 mm^3 (day 1). The tumor volume was measured on day 22. Each group consisted of 6 mice. Data are shown as means ± SD. * $P < 0.05$ compared with vehicle-treated mice. (b) The percentage of tumor growth inhibition (%TGI). Each group consisted of 6 mice. Antitumor activity was calculated as %TGI at day 22. Data are shown as means ± SD. * $P < 0.05$ compared with %TGI between lenvatinib at 100 mg/kg and sorafenib at 300 mg/kg-treated groups. (c) Comparison of antitumor activity with antiangiogenesis activity with lenvatinib treatment compared with sorafenib. To analyze the relationship between antitumor activity (%TGI) and antiangiogenesis activity (percentage of microvessel inhibition, %MVI), average %TGI and %MVI values for each dose of either lenvatinib or sorafenib-treated groups were plotted on the X- and Y-axes, respectively. %TGI and %MVI was determined as described in Materials and Methods. Analysis of covariance (ANCOVA) for %TGI was examined by using %MVI as a covariate ($P<0.05$)

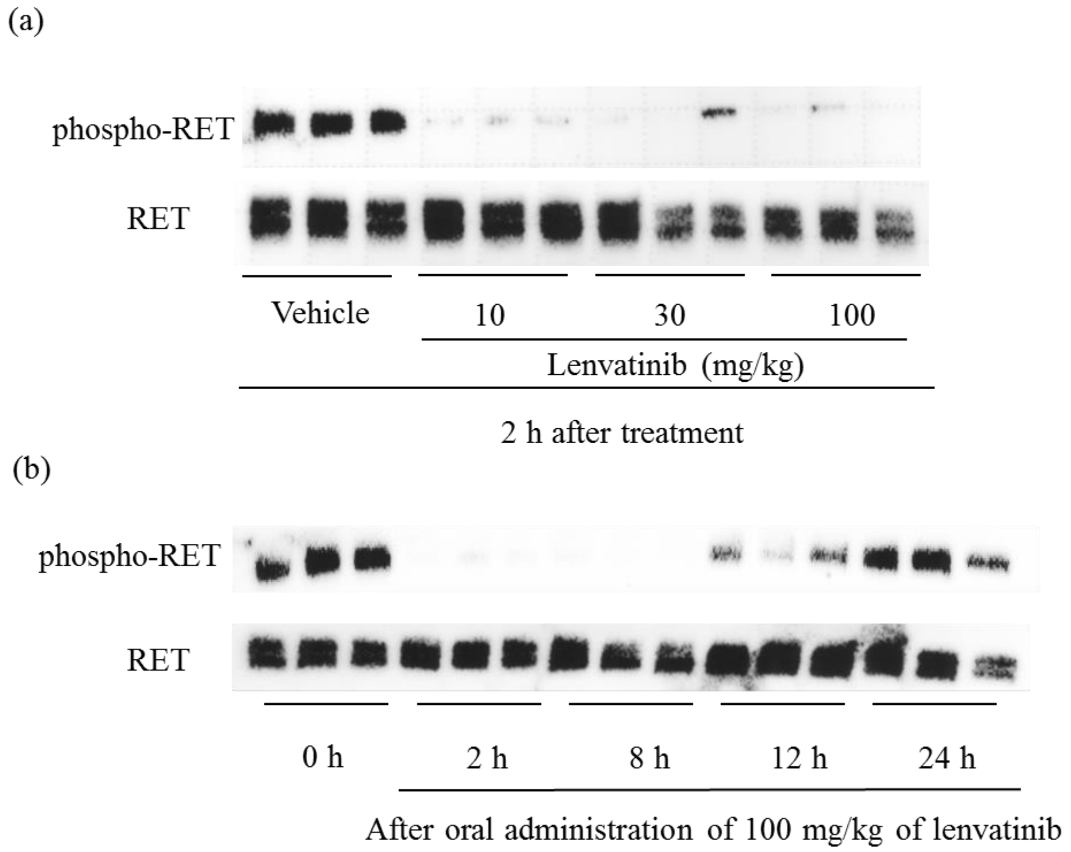

Supplementary Fig. S8 Effect of lenvatinib on the phosphorylation of RET in human medullary thyroid cancer TT xenografts in nude mice. Western blot analysis of the phosphorylation of RET in the TT xenograft model was performed. Nude mice bearing TT xenografts were treated once orally with either vehicle or lenvatinib at the indicated doses, when tumor volumes reached approximately 100-300 mm^3. Each group consisted of 3 mice. Tumors were collected at the indicated times after administration, lysed with lysis buffer, and then used for western blot analysis as described in the Materials and Methods (a) Phosphorylation of RET 2 h after treatment with lenvatinib. (b) Phosphorylation of RET at the indicated times after treatment with lenvatinib. Representative images are shown.
